# Prosthetic Joint Infection due to* Mycobacterium avium-intracellulare* in a Patient with Rheumatoid Arthritis: A Case Report and Review of the Literature

**DOI:** 10.1155/2017/8682354

**Published:** 2017-02-09

**Authors:** Nicholas E. Ingraham, Brenton Schneider, Jonathan D. Alpern

**Affiliations:** ^1^Department of Internal Medicine, University of Minnesota, Minneapolis, MN, USA; ^2^Department of Internal Medicine, Hennepin County Medical Center, Minneapolis, MN, USA; ^3^Department of Infectious Disease, University of Minnesota, Minneapolis, MN, USA

## Abstract

Nontuberculous mycobacteria (NTM) are a rare cause of prosthetic joint infections (PJI). However, the prevalence of NTM infections may be increasing with the rise of newer immunosuppressive medications such as biologics. In this case report, we describe a rare complication of immunosuppressive therapies and highlight the complexity of diagnosing and treating PJI due to NTM. The patient is a 79-year-old Caucasian male with a history of severe destructive rheumatoid arthritis on several immunosuppressive agents and right hip osteoarthritis s/p total hip arthroplasty 15 years previously with several complex revisions, presenting with several weeks of worsening right hip and abdominal pain. A right hip CT scan revealed periprosthetic fluid collections. Aspiration of three fluid pockets was AFB smear-positive and grew* Mycobacterium avium*-*intracellulare*. The patient was deemed a poor surgical candidate. He underwent a limited I&D and several months of antimycobacterial therapy but clinically deteriorated and opted for hospice care. PJI caused by NTM are rare and difficult to treat. The increased use of biologics and prosthetic joint replacements over the past several decades may increase the risk of PJI due to NTM. A high index of suspicion for NTM in immunosuppressed patients with PJI is needed.

## 1. Introduction

A 79-year-old man with a history of a right total hip arthroplasty (THA) performed 15 years prior to admission was admitted with 3 weeks of right hip pain. His history was notable for severe rheumatoid arthritis (RA), Alzheimer's dementia, coronary artery disease, and several arthroplasty revisions, which were performed 10 and 5 years prior to admission due to dislocations. His RA had been treated with prednisone 15 mg daily, leflunomide 10 mg daily, and infliximab. Six months prior to admission infliximab was replaced with abatacept 500 mg every month.

**Figure 1:**
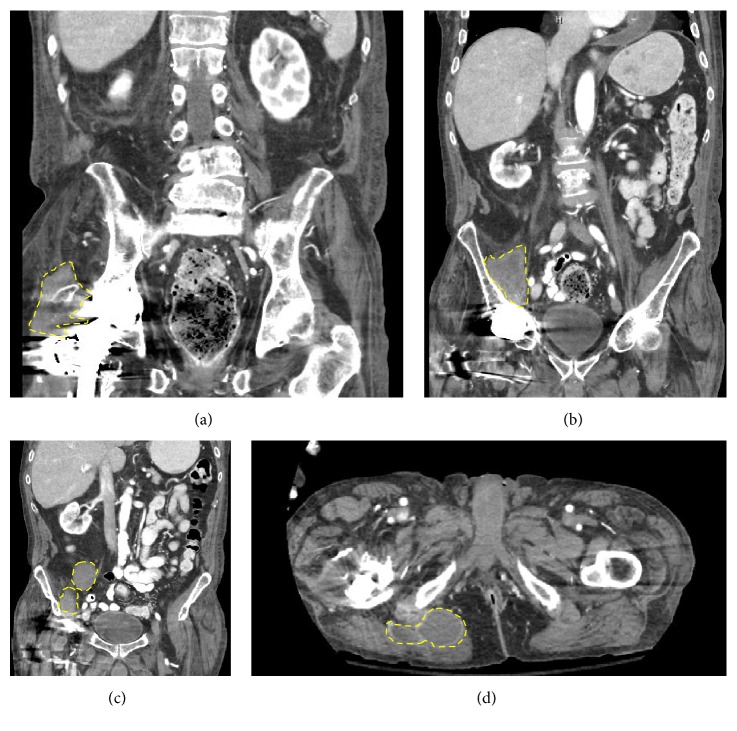
CT abdomen. (a) Coronal cross section demonstrating a right hip complex fluid collection. (b) Right posterior iliac fossa fluid collection. (c) Right anterior iliac fossa fluid collections. (d) Transverse section demonstrating right buttock fluid collection.

At presentation, vital signs were normal. BMI was 22.9 kg/m. He had right lower quadrant tenderness and pain with internal/external rotation of the right lower extremity. His WBC count was within normal limits. The CRP was 5.2 mg/L (normal < 3 mg/L) and ESR was 28 mm/h (normal < 15 mm/h). A CT scan of the abdomen and pelvis revealed complex fluid collections involving the right hip, buttock, and iliac fossa (see Figure 1). A joint aspirate contained 2734 cells/*μ*L WBC with 89% neutrophils. Aspirates from his joint and fluid collections were sent for Gram stain and culture, AFB smear and culture, and fungal cultures. Blood cultures were not sent. The AFB smears from each joint and fluid collection resulted positive for acid fast bacilli. The interferon gamma release assay (Quantiferon Gold) was indeterminate. HIV was not checked. His immunosuppression was adjusted, and leflunomide and abatacept were replaced with methotrexate and hydroxychloroquine. On day 14, mycobacterial cultures speciated as* Mycobacterium avium-intracellulare *(MAI). Rifampin, ethambutol, and clarithromycin were started.

A staged arthroplasty revision was not considered due to his comorbidities. Instead, he underwent extensive incision and drainage (I&D) of the joint and surrounding tissues, with percutaneous drain placement into the fluid collections. On discharge, he was continued on antimycobacterials for chronic suppression. After 4 months of therapy with ethambutol, clarithromycin, and rifampin, he continued to have positive AFB smears from the surgical site and percutaneous drains. After several months of declining quality of life, he was placed on hospice and died 6 months after initial presentation.

## 2. Discussion

MAI (*M. avium* and* M. intracellulare*) are slow growing mycobacteria that typically cause human disease in the form of pulmonary infection among immunocompetent hosts, disseminated infection in the setting of HIV/AIDS, and cervical lymphadenitis [1]. Mycobacteria are a rare cause of prosthetic joint infection (PJI), making up 0–0.6% of cases, with the majority of these caused by* M. tuberculosis* [2, 3]. In one retrospective study of PJI caused by rapidly growing mycobacteria (RGM), only eight cases were identified over a 38-year time period, with the majority of cases occurring in immunocompetent hosts [4]. In contrast, PJI due to MAI occurs most often in immunocompromised hosts and is significantly rarer (see Table 1) [2, 5–10]. To the best of our knowledge, this is the 7th reported case of PJI due to MAI and the first case in which the predisposing factor was immunosuppression due to antirheumatic medications.

In this patient, mycobacteria were likely introduced during a THA revision and persisted for years prior to disease onset in the setting of exogenous immunosuppression [11]. TNF-alpha antagonists, particularly infliximab, predispose patients to mycobacterial infections by blocking macrophage activation and intracellular killing [12]. The patient had risk factors both for PJI in general (RA, exogenous immunosuppressive medications) and for mycobacterial infection specifically, due to the use of multiple disease-modifying antirheumatic drugs (DMARDs). The use of oral corticosteroids and certain DMARDs such as TNF-alpha inhibitors and leflunomide increases the risk of both tuberculous and nontuberculous mycobacterial infections among patients with RA [13]. Abatacept has been associated with tuberculosis; however whether or not the drug is a risk factor for mycobacterial infection is unclear [14].

Making the diagnosis of NTM PJI requires obtaining mycobacterial cultures from clinical specimens in immunosuppressed patients at risk for NTM. Optimal management relies on experience from pulmonary MAI and PJI due RGM. A prolonged course of three drugs (macrolide, ethambutol, and rifampin) is recommended, in addition to surgical debridement or surgical excision [15]. Susceptibilities should be obtained to guide therapy. A case series of patients with PJI due to RGM suggested that resection arthroplasty with appropriate antimycobacterial drugs resulted in the best chance for cure. If debridement with prosthesis retention is performed, prolonged and indefinite antimycobacterial drugs are likely needed to prevent relapse [2, 4, 16]. Similar to PJI caused by rapid-growing NTM species, the prognosis for slow growing NTM PJI is variable (see Table 1).

In conclusion, MAI is a rare cause of PJI, occurring almost exclusively in immunocompromised patients. Among patients with PJI taking DMARDs or other immunosuppressive biologic medications, a high index of suspicion is needed for opportunistic infections such as MAI. Ensuring multiple intraoperative specimens is sent for AFB smear/culture is imperative for diagnosis.

## Figures and Tables

**Table 1:** Prosthetic joint infections caused by *Mycobacteriumavium* species.

Citation	Age/gender	Pathogen	Site	Reason for arthroplasty	Predisposing factors	Time to symptom onset	Surgical treatment	Medical treatment	Outcome
[5]	67/F	MAC	Hip	DJD	Renal transplant on cyclosporine, prednisone	15 yrs after arthroplasty 13 yrs after transplant	I&D, removal of prosthesis, spacer placement Repeat I&D and spacer removal for sterile wound drainage 4 months post-op	Azithromycin, ethambutol, rifabutin	Planned reimplantation at 6 months and antimycobacterial coverage for total of 18 months

[6]	41/M	MAI (disseminated)	Knee	Salmonella septic arthritis	Suspected underlying immunodeficiency syndrome Polymyositis on prednisolone	15 mo after TKR (MRSA isolated after TKR)	Repeat debridements	Multiple antibiotic regimens	Died due to sepsis with MRSA, *E.coli*, *K.pneumoniae*, MAI, *Candida* 7 months after initial MAI culture

[7]	NR	*M. avium*	Hip	Osteonecrosis	Heart transplant on cyclosporine, prednisone	NR	None	Ethambutol, rifampin, isoniazid	Doing well at time of follow-up (time NR)

[8]	20/M	MAC (disseminated)	Bilateral hips	Perthes disease	AIDS	20 yrs after arthroplasty 4 yrs after HIV diagnosis	Right hip resection arthroplasty	Ciprofloxacin, clarithromycin, rifampicin, clofazimine	Died 5 months post-op

[9]	73/M	MAI (disseminated)	Knee	DJD	Multiple myeloma on lenalidomide, dexamethasone RA on methotrexate Previous pulmonary and olecranon MAC infections	3 yr after chemotherapy, 1 yr after arthroplasty	Resection arthroplasty (reimplantation 7 mo post-op)	Clarithromycin, ethambutol	Doing well at 7 years of follow up

[10]	39/M	MAI (disseminated)	Hip	Osteonecrosis	Renal transplant	NR	Resection arthroplasty	Azithromycin, ethambutol > 12 mo; did not tolerate rifabutin	No recurrence on ethambutol, clarithromycin; reimplantation of prosthesis not performed

DJD, degenerative joint disease; I&D, incision and drainage; MAC, *Mycobacteriumavium* complex; MAI, *Mycobacteriumavium*-*intracellulare*; mo, months; MRSA, methicillin-resistant *S. aureus*; NR, not reported; PCP, *Pneumocystis carinii* pneumonia; TKR, total knee replacement; yrs, years.
